# Prevalence of and risk factors for perioperative arrhythmias in neonates and children after cardiopulmonary bypass: continuous holter monitoring before and for three days after surgery

**DOI:** 10.1186/1749-8090-5-85

**Published:** 2010-10-18

**Authors:** Lars Grosse-Wortmann, Suzanna Kreitz, Ralph G Grabitz, Jaime F Vazquez-Jimenez, Bruno J Messmer, Goetz von Bernuth, Marie-Christine Seghaye

**Affiliations:** 1 Department of Paediatric Cardiology, Aachen University Hospital, Aachen, Germany; 2 The Labatt Family Heart Centre at The Hospital for Sick Children, The University of Toronto, Canada; 3 Department of Cardiac Surgery, Aachen University Hospital, Aachen, Germany

## Abstract

**Background:**

A comprehensive evaluation of postoperative arrhythmias following surgery for congenital heart disease by continuous Holter monitoring has not been carried out. We aimed, firstly, to establish the time course of pre- and early postoperative arrhythmias by beat-to-beat analysis following cardiopulmonary bypass and, secondly, to examine which surgical procedures present risk factors for specific arrhythmias.

**Methods:**

494 consecutive patients, including 96 neonates, were studied with serial 24-hour Holter electrocardiograms before as well as uninterruptedly during the first 72 hours after surgery and prior to discharge.

**Results:**

Within 24 hours of surgery 59% of the neonates and 79% of the older children developed arrhythmias. Junctional ectopic tachycardia occurred in 9% of neonates and 5% of non-neonates and ventricular tachycardia in 3% and 15%, respectively.

For neonates, male sex and longer cross-clamping time independently increased the risk for arrhythmias (odds ratios 2.83 and 1.96/minute, respectively). Ventricular septal defect repair was a strong risk factor for junctional ectopic tachycardia in neonates and in older children (odds ratios 18.8 and 3.69, respectively). For infants and children, older age (odds ratio 1.01/month) and closure of atrial septal defects (odds ratio 2.68) predisposed to arrhythmias of any type.

**Conclusions:**

We present the largest cohort of neonates, infants and children that has been prospectively studied for the occurrence of arrhythmias after cardiac surgery. Postoperative arrhythmias are a frequent and transient phenomenon after cardiopulmonary bypass, provoked both by mechanical irritation of the conduction system and by humoral factors.

## Background

Arrhythmias are common in the early postoperative period after cardiac surgery for congenital heart disease[1-3]. Although transient and treatable in most cases, they are the cause of substantial morbidity and mortality during a vulnerable phase of hemodynamic instability.

Thus far the overall incidence and risk factors of transient early postoperative arrhythmias in neonates and children undergoing cardiac surgery have only been addressed in a limited number of studies,[3,4] each using overhead bedside monitoring. While this method is sensitive enough for sustained and hemodynamically significant arrhythmias, shorter or more subtle rhythm disorders that may still reflect electrical instability of the myocyctes and a propensity to develop hemodynamically significant rhythm disturbances, may remain undetected. Furthermore, the exact postoperative timing when arrhythmias are most likely is unknown and may provide a clue to their etiology. With this study, we sought to establish the prevalence and time course of early postoperative arrhythmias following cardiopulmonary bypass (CPB), using continuous dual lead Holter monitoring. We also aimed to examine whether certain surgical procedures present risk factors for specific arrhythmias.

## Methods

Following approval by our institution's Human Ethical Committee and the parents' informed consent, 494 consecutive patients undergoing CPB for corrective surgery of congenital cardiac defects were enrolled prospectively between November of 1993 and February of 2000: 96 neonates, aged 1-28 days, with a median age of 9 days, and 398 infants and children between 29 days and 18 years of age, with a median age of 3.7 years. Neonates were analyzed separately from infants and older children because they were homogeneous with regards to age and surgical procedure (88.5% underwent an arterial switch operation for transposition of the great arteries). Norwood operations or Damus-Kaye-Stansel procedures were not performed in Aachen during the study era. Patient characteristics and the surgical procedures performed are summarized in Table 1.

**Table 1:** Patient characteristics

	all (n = 494)	neonates (n = 96)	infants and children (n = 398)	P
Age in months	12.2 (0.0-218.4)	0.2 (0.0-0.9)	24.4 (1.1 months to 18 years)	< 0.0001
cardiopulmonary bypass in min	65 (24-264)	50 (33-165)	71 (24-264)	< 0.0001
cardiocirculatory arrest used in	352 (71.3%)	96 (100%)	256 (64.3%)	
duration if cardiocirculatory arrest was used in min	59 (14-158)	59 (21-127)	58 (14-158)	0.01
aortic clamping time in min	61 (2-177)	62 (23-125)	61 (2-177)	0.05
Male	291 (58.9%)	67 (70.0%)	224 (56.3%)	0.04
repair of atrial septal defect	213 (43.1%)	87 (90.6%)	126 (31.7%)	< 0.0001
repair of isolated atrial septal defect of secundum type	56 (11.3%)	2 (2.1%)	54 (13.6%)	0.07
repair of incomplete atrioventricular septal defect	31 (6.3%)	1 (1.0%)	30 (7.5%)	0.3
Repair of complete atrioventricular septal defect	26 (5.3%)	0	26 (6.5%)	
repair of ventricular septal defect (all types)	189 (38.3)	21 (21.9%)	168 (42.2%)	0.002
repair of isolated subarterial ventricular septal defect	51 (10.3%)	0	51 (12.8%)	
repair of tetralogy of Fallot	69 (13.0%)	1 (1.0%)	68 (17.1%)	0.01
right ventricular outflow tract surgery	107 (21.7%)	1 (1.0%)	106 (26.6%)	< 0.0001
repair of subaortic stenosis	13 (2.6%)	0	13 (3.3%)	
repair of total anomalous pulmonary venous return	13 (2.6%)	2 (2.1%)	11 (2.8%)	0.9
Fontan operation and its modifications *	25 (5.1%)	0	25 (6.3%)	
arterial switch operation	89 (18.0%)	85 (88.5%)	4 (1.0)	< 0.0001
miscellaneous operations	102 (20.6%)	7 (7.3%)	95 (23.9%)	0.009

Patients who underwent closure of a secundum type atrial septal defect are also included in those with atrial septal defect repair. Patients with repair of atrioventricular septal defect include those with the complete and the incomplete type of the defect. The subgroup of subarterial ventricular septal defects is also analysed in the group of ventricular septal defects. Patients with repair of tetralogy of Fallot are also included in the cohort of patients who underwent right ventricular outflow tract surgery.

*including 2 patients with a classic Fontan repair and 29 with total cavopulmonary connections

### Anesthesia and Cardiopulmonary Bypass

In all patients, conventional general anesthesia was performed. A standardized bypass protocol, including the administration of dexamethasone for cerebral edema prophylaxis and inflammation control as well as heparin was employed. After aortic and either right atrial or bicaval cannulation CPB was instituted with a perfusion index of 2.7 l/min/m^2 ^of body surface area. Hypothermia was obtained by cooling the priming solution within the extracorporeal circuit and the circulating blood volume using a heat exchanger. Aortic cross clamping was undertaken and cardioplegia induced by a single aortic injection of 30 ml/kg of 4°C cold Bretschneider solution. If necessary, deep hypothermic arrest was established. Where indicated, the surgical procedure was continued under low-flow perfusion (25% of the calculated initial perfusion rate). Re-warming was carried out under full-flow conditions. Neutralization of heparin was achieved by equivalent doses of protamine sulfate. All patients received sodium nitroprusside for vasodilatation during rewarming (0.5 to 2 μg/kg/minute). If necessary, catecholamines (epinephrine, dobutamine) were administered while weaning the patient from CPB. All patients left the operating theatre with temporary transcutaneous atrial and ventricular pacemaker electrodes *in situ *which were removed between eight and ten days postoperatively.

### Postoperative care

Postoperatively, the catecholamine and vasodilatory regimen was adapted to the particular hemodynamic situation. Partial pressure of oxygen and of carbon dioxide, oxygen saturation and electrolytes were monitored and kept within the normal range: pH 7.40-7.50, partial pressure of oxygen and oxygen saturation according to the type of repair, partial pressure of carbon dioxide 35-40 mm of mercury, sodium 135-145 mmol/l, potassium 3.8-4.5 mmol/l, calcium 2.1-2.6 mmol/l, serum ionized calcium 1.14-1.30 mmol/l, and magnesium 0.8-1.3 mmol/l. Episodes of junctional ectopic tachycardia (JET) were treated by cooling and, if necessary, atrial overdrive pacing. Complete heart block was managed by temporary or, if persistent past postoperative day 7, permanent, pacing.

### Assessment and definitions of arrhythmias

24-hour Holter electrocardiograms were performed before the operation (with the exception of patients referred as an emergency), continuously for the first 72 hours after the operation and again between postoperative day 10 and 15, which in most cases was shortly prior to discharge. Standard 12-lead electrocardiograms were also recorded before and 4, 24, 48, 72 hours after the operation as well as before discharge. When P waves could not be clearly identified in the Holter recording and in the surface tracings, atrial electrocardiograms performed, using the atrial pacemaker lead. 24-hour Holter electrocardiograms were analyzed by manual analysis, using the Medilog Electrocardiogram-Analysis System (Oxford Instruments GmbH, Wiesbaden, Germany). Postoperative arrhythmias were diagnosed according to the following definitions:[5]

*Supraventricular extrasystoles (SVE): *Premature atrial or junctional contractions when occurring more often than 49 beats/24 hours.

*Ventricular extrasystoles (VE): *Premature ventricular contractions if their total number exceeded 49 beats/24 hours.

*Supraventricular tachycardia (SVT)*: Series of 3 or more repetitive supraventricular beats with an abnormally rapid atrial rate for age.

*Ventricular tachycardia (VT)*: Series of 3 or more repetitive excitations originating from one of the ventricles. The QRS complexes are different from the patient's usual QRS morphology with a prolonged duration for age.

*Junctional rhythm (JR)*: Junctional escape rhythm with normal QRS morphology at a rate not exceeding the maximum normal junctional escape rate for age (50 to 80 beats/min up to 3 years, and 40 to 60 beats/min over 3 years) and slower than the atrial escape rhythm (80 to 100 beats/min up to 3 years, and 50 to 60 beats/min over 3 years).

*Accelerated junctional rhythm (AJR)*: Supraventricular rhythm with normal QRS complexes and no preceding P wave, with a ventricular rate faster than the normal junctional escape rate but not exceeding the maximum normal sinus rate for age at rest.

*JET*: AJR with a QRS rate exceeding the maximum normal sinus rate of age.

First-degree atrioventricular block (AVB) was considered physiological and therefore not analyzed.

Atrial flutter or fibrillation were not encountered during the study.

### Statistics

Demographic data and clinical results are expressed as the median values and ranges. Period prevalences are expressed as frequencies (%). Assuming non-normal distribution of the data, non-parametric tests were used for comparison of prevalences between patient groups and at different time points. Differences between prevalences were regarded as significant if the t-test's p value was less than 0.05. The presence of risk factors in the various types of arrhythmias was analyzed using the Fisher exact test. The risk factors were entered into a stepwise multivariate logistic regression model. From the first step of the logistic regression, factors with a p value less than 0.1 were entered in the final regression model in which p values of less than 0.05 indicated statistical significance.

All authors read and approved the final manuscript.

## Results

### Incidence of arrhythmias

#### Neonates

**Figure 1:**
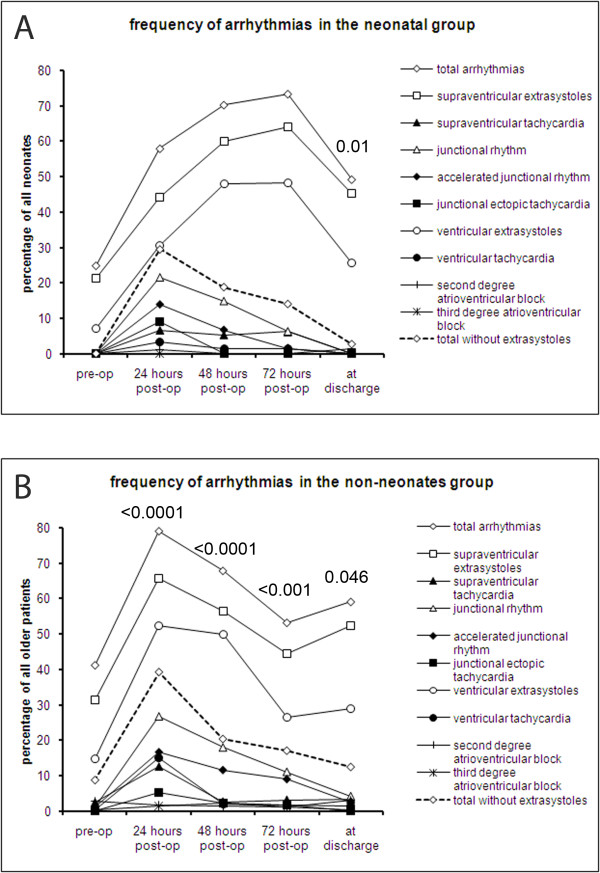
**Period prevalences of arrhythmias**. The period prevalences of all arrhythmias as well as of the individual types of arrhythmias preoperatively, continuously during the first 72 hours after cardiopulmonary bypass and prior to discharge in A) neonates and B) non-neonates. The p values shown pertain to the comparison of the overall prevalence arrhythmias during the respective period with the preceding Holter monitoring period. No p values are shown when the difference was non-significant.

Of the 96 neonates, n = 51, n = 38, n = 30, and n = 38 neonates had Holter electrocardiograms at 24, 48, 72 hours and prior to discharge, respectively. In 40 neonates (42%), Holter studies were performed at at least three out of the five time points. One third of neonates (n = 28) had had a preoperative Holter study, anda percentage of these had postoperative follow-up studies at the various time points (n = 28, n = 21, n = 14, and n = 20 within 24, 48, 72 hours and prior to discharge, respectively). The preoperative period prevalences of arrhythmias during 24 hour Holter monitoring was 25% (Figure 1A). The postoperative prevalences did not differ significantly from the preoperative ones. The prevalence of arrhythmias peaked at 73% after 72 hours (p = 0.4 versus preoperative prevalence). Before discharge, arrhythmias were detected in 49% (p = 0.7 versus preoperative prevalence).

Supraventricular and ventricular extrasystoles were the only types of arrhythmias seen preoperatively (SVE and VE in 21% and 7%, respectively) and constituted the most common arrhythmias at all time points (Figure 1A). Their period prevalence peaked between 48 and 72 hours after surgery at 64% for SVE and 48% for VE, respectively, although the increase was not significant (presumably because of small patient numbers). All other types of arrhythmias were most frequent within the first 24 hours after CPB: SVT, 7%; AJR, 14%; JET, 9%; VT, 3%. Their prevalence either decreased or remained unchanged until discharge. The postoperative prevalences were not significantly different from the preoperative ones. Second degree AVB affected 1.1% of patients within 24 hours and 1.4% prior to discharge. None of these patients had had a preoperative Holter. Complete heart block did not occur in any of the neonates. Antiarrhythmic drugs were not necessary in any of our patients.

#### Infants and children

Among the infants and children, n = 287 (72.1%), n = 378 (95.0%), n = 287 (72.1%), n = 241 (60.6%) and n = 282 (70.9%) received Holter monitoring at the various time points between admission and discharge. A minimum of three out of five possible Holter examinations were conducted in 301 infants and children (75.6%). 251 (63.1) had Holter studies within 24 hours after CPB as well as prior to discharge. Prior to surgery, 41.2% of patients older than 28 days experienced arrhythmias (Figure 1B). Following CPB, the prevalence of arrhythmias increased and peaked within 24 hours after surgery at 79.1% (p < 0.0001). Thereafter, the portion of patients with arrhythmias declined to 53.2% at 72 hours (p < 0.0001 as compared to 48 hours postoperatively), and rose again to 59.1% prior to discharge (p = 0.046). This prevalence was significantly higher than prior to surgery (p < 0.0001). Supraventricular and ventricular extrasystoles were the most common arrhythmias at all time points with a peak prevalence in the first 24 hours after surgery (65.8%, p = 0.2, and 52.3%, p = 0.0001, compared to preoperatively, Figure 1B). Their prevalence decreased thereafter. At discharge, SVEs were not significantly more frequent than preoperatively (31.3% vs. 52.4%, p 0.3). The prevalence of VEs, however, remained elevated at the time of discharge (28.9% vs. 14.8%, p = 0.03) as compared to preoperative values. The prevalence of other types of arrhythmias also peaked within 24 hours after surgery (Figure 1B). At that time point, the period prevalences were 12.7% for SVT, 16.7% for AJR, 5.4% for JET, and 15.2% for VT (p < 0.0001 versus preoperative prevalence for all types of arrhythmias). The prevalences of these types of arrhythmias reached preoperative values before discharge.

Second degree AVB was not detected before the operation and occurred in 1.9% of all infants and children within 24 hours of CPB (non-significant versus preoperatively). The prevalence was 1.1% after 72 hours and increased, albeit not significantly, to a maximum of 3.1% prior to discharge. Complete heart block increased from 0.4% before the operation to 2.4% 48 hours postoperatively (p = 0.03). 37.5% of patients with second degree and all patients with third degree AVB required definitive pacemaker therapy.

Overall, transient pacemaker therapy was necessary in 19.9% 24 hours after the operation. 6.2% of those in need of temporary pacing patients required definitive pace maker implantation before discharge.

#### Comparison neonates - infants and children

Preoperatively, there was no significant difference in period prevalence between the two groups (p = 0.11). Within 24 hours of CPB, however, arrhythmias were more frequent in infants and older children than in neonates (79.1% vs. 58.0%, p < 0.0001). In contrast, 72 hours after surgery, arrhythmias were less frequent in the older children than in neonates (53.2% vs. 73.4%, p = 0.0033). Prior to discharge, there was once again no significant difference in the prevalence of arrhythmias between the two age groups (p = 0.15). The peak period prevalence for the total number of arrhythmias as well as for extrasystoles was not different in magnitude between the two groups (73.4% and 79.1% in neonates and in older children, respectively, p = 0.73), albeit occurring later in neonates than in older children. Within 24 hours after surgery, which marked the period of peak prevalence of all types of arrhythmias other than supraventricular and ventricular premature contractions in both patient groups, there was no significant difference between them in the prevalence of JR (21.6%, vs. 26.8% p = 0.7), AJR (14.0%, vs 16.7%. p = 0.6), JET (9.1% vs. 5.4%, p = 0.2), VT (3.4% vs. 15.2%, p = 1.0).

#### Risk factors for arrhythmias

Risk factors and their odds ratios are summarized in Table 2 for neonates and Table 3 for infants and children. Among neonates, boys and patients with long aortic cross clamp times or shorter CPB durations were at risk for early postoperative arrhythmias. Closure of a ventricular septal defect (VSD) predisposed to the development of JR, AJR, JET and VT. The arterial switch operation did not emerge as a risk factor for the development of arrhythmias in the early postoperative period.

**Table 2:** Risk factors for early postoperative arrhythmias in neonates

risk factor	Any	supra-ventricular extra-systoles	supra-ventricular tachy-cardia	junctional rhythm	accelerated junctional rhythm	Junctional ectopic tachy-cardia	ventricular extra-systoles	ventricular tachycardia
male sex	2.81 (1.03-7.66) p 0.04	3.19 (1.12-9.09) p 0.03						

cardiopulmonary bypass per min	0.96 (0.94-0.99) p 0.04						0.97 (0.92-0.99) p 0.01	

aortic clamping time per min	1.06 (1.01-1.12) p 0.03							

Ventricular septal defect repair				6.86 (2.14-21.97) p 0.001	8.96 (2.38-33.78) p 0.001	18.82 (3.36-105.32) p 0.001	13.67 (2.95-63.31) p 0.001	

Odds ratios, with 95% confidence intervals in brackets, are presented only if significant in a multivariate analysis model of all risk factors

**Table 3:** Risk factors for early postoperative arrhythmias in non-neonates

risk factors	Any	supra-ventricular exta-systoles	supra-ventricular tachy-cardia	junctional rhythm	accelerated Junctional rhythm	junctional ectopic tachy-cardia	ventricular extra-systoles	ventricular tachy-cardia
Age per month	1.01 (1.00-1.01) p 0.03	1.01 (1.00-1.01) p 0.003					1.01 (1.01-1.02) p < 0.0001	1.01 (1.01-1.02) p < 0.0001

cardiocirculatory arrest per minute					1.03 (1.01-1.04) p < 0.0001			

Atrial septal defect repair	2.68 (1.41-5.12) p 0.003	2.29 (1.45-3.95) p 0.0007		2.39 (1.31-4.37) p 0.003				

Secundum atrial septal defect repair				0.19 (0.06-0.58) p 0.004				0.19 (0.04-0.78) p 0.02

Ventricular septal defect repair						3.69 (1.36-10.01) p 0.01		

subarterial ventricular septal defect repair					2.54 (1.13-5.70) p 0.02		2.58 (1.32-5.05) p 0.006	

subaortic stenosis repair						5.93 (1.05-29.87) p 0.04		

Fontan operation or related procedures				3.53 (1.50-8.31) p 0.004	3.6 4 (1.47-9.01) p 0.005			

Odds ratios, with 95% confidence intervals in brackets, are presented only if significant in a multivariate analysis model of all risk factors

Among infants and children, those who had arrhythmias prior to surgery were more likely to experience rhythm disorders during the first 24 hours after surgery than those without preoperative arrhythmias (68.8% vs. 48.9%, p 0.02). Older age predisposed to the development of arrhythmias, in particular SVE, VE and VT. Gender, duration of CPB and aortic cross clamp time did not manifest as risk factors for postoperative arrhythmias. Ventricular septal defect repair was a risk factor for JET and Fontan type procedures predisposed to JR and AJR. Surgery for atrioventricular septal defects (AVSDs), repair of total anomalous pulmonary venous connection, tetralogy of Fallot or other forms of right ventricular outflow tract obstruction were not risk factors for the development of arrhythmias. Due to the small number of patients with AVB, logistic regression analysis for its risk factors was not possible. However, complete heart block occurred more often after repair of complete AVSD (8.0% vs. 1.7%, p = 0.04) than after other procedures.

## Discussion

Previous studies of postoperative arrhythmias after surgery for congenital heart disease focused either on specific types of arrhythmias, for example JET, after a variety of operations, or on the arrhythmias associated with a limited number of distinct cardiac lesions or surgical procedures, such as early and late SVT following Fontan operations[6-10]. We report the hitherto largest cohort of consecutive patients that were assessed for the occurrence of all types of arrhythmias following CPB. During the immediate postoperative period, which tends to be a time of heightened vulnerability after cardiac surgery, we were able to conduct Holter studies in almost all infants and children. Previous studies used overhead bedside monitoring, either for one day[3] or for an extended period of time[11].

Up to now, no study has provided comprehensive information derived from beat-to-beat dual lead continuous Holter monitoring on the prevalence and type of arrhythmias in this patient population. In a study of 100 patients after cardiac surgery, Valsangiacomo et al. identified arrhythmias in 48% within 1 day of cardiac surgery[3]. Pfammatter and colleagues found an arrhythmia prevalence of 27% in 310 patients after CPB. Delaney et al. reported only on those arrhythmias in their patients that necessitated intervention which made up 15% of their cohort[2]. In in patients we studied, the peak prevalence of arrhythmias of 73.4% and 79.1% in the two groups was higher than those previously reported, which may reflect a more sensitive method of detection, especially of extrasystoles, using Holter instead of bedside monitoring. When we disregarded these usually benign rhythm abnormalities, the peak prevalences dropped to 29.6% and 38.9% in neonates and older children, respectively (Figure 1), which is in keeping with the previous literature and with the expectation that "non-extrasystole" arrhythmias that are more clinically significant will rarely remain undetected [3,11]. Nevertheless, more benign arrhythmias may reflect an electrical instability of the cardiac myocytes and a propensity to develop more severe forms of tachyarrhythmias during the patient's postoperative course. In the neonatal group, all significant types of arrhythmias were nearly absent at the time of discharge and JET was confined to the immediate 24 hours after CPB. In the older children group, 12.5% of "non-premature beats" arrhythmias were detected on the last Holter electrocardiogram and JET must be expected as late as 72 hours after surgery.

Our results confirm that early postoperative arrhythmias are a mainly transient phenomenon after surgery[3,11]. This is especially true for the more detrimental types of arrhythmias, other than premature contractions. Nevertheless, in infants and children, arrhythmias remained significantly more frequent prior to discharge than before the operation. This finding suggests that so-called late arrhythmias after surgery for congenital heart disease do not always develop de-novo as a late complication of scarring or ventricular failure, but may be a heritage from early postoperative arrhythmias at least in a proportion of patients. None of the previous studies investigated the preoperative prevalence of arrhythmias and their effect on postoperative arrhythmias. In this study, we started Holter monitoring 24 hours prior to surgery. While our results confirm the expected finding that the prevalence of arrhythmias increases after CPB, they also revealed that children with pre-operative arrhythmias were significantly more likely to have postoperative arrhythmias. These pre-operative arrhythmias were benign in nearly all cases and may reflect a preceding injury to the conduction system, either from previous surgery, or from chronic hypoxemia or chamber dilation with congestive heart failure.

Surgical manipulation of the conduction system[12,13] has been identified as a cause of postoperative arrhythmias, and the site of mechanical irritation has been linked to the type of arrhythmia encountered, both by other investigators as well as in this series[12,13]. For instance, SVT was common in both groups, likely a result of cannulation of the right atrium for CPB and surgical access via the atria. Another example is the association of closure of a VSD with a variety of junctional neonatal arrhythmias, including JET. In older children, repair of a VSD, but not if the defect was remote from the atrioventricular node, such as isolated subarterial VSDs, was an independent predictor of JET. Therefore, surgical injury to the conduction system appears to play an important role in the development of JET, as has been speculated by others[13-15]. On the other hand, repair of complete AVSD, despite involving the atrioventricular junction, was not associated with JET. This is in contrast to a report by Batra and colleagues[8], and by Delaney et al. who identified the same intervention as a risk factor for symptomatic arrhythmias, most of them JET[2]. In our series a relatively low number of 26 complete AVSD repairs may have been too few to unmask an independent association with JET.

Junctional ectopic tachycardia typically manifests two to eight days after CPB. The reported incidence after repair of congenital heart defects varies widely, with the largest study in children stating 5.6%, which is similar to our findings[2,3,8,16]. Hoffman and colleagues found younger age to be a strong risk factor for the development of JET. In our cohort, it was almost twice as frequent among neonates as among older children.

Other factors than mechanical injury likely contribute to the development of junctional arrhythmias, as indicated by the increased risk for junctional tachycardias following interventions that were remote from the atrioventricular node (including resection of subaortic stenosis and atrial septal defect closure, Table 2). Myocardial injury as a result of ischemia and reperfusion during CPB has been suggested as a cause of arrhythmias after cardiac surgery[2,3,17]. Consequently, markers of myocardial damage, such as troponin, have been used to predict postoperative arrhythmias[18]. Inflammatory mediators that are released in the early postoperative period appear to contribute to the development of arrhythmias by altering the myocytes' membrane potential,[19] and possibly facilitating micro-reentry within the atrium and the atrioventricular node. Histamine, which is liberated as part of the postoperative inflammatory response, has been shown to exhibit pro-arrhythmogenic properties[20]. A protective effect against arrhythmias has been attributed to estradiol which inhibits the release of inflammatory cytokines[21]. Estradiol is higher in newborn girls than in boys, which may explain why, in our study, newborn boys were at greater risk for arrhythmias.

The majority of neonates underwent an isolated arterial switch operation for simple transposition of the great arteries, avoiding direct injury to the conduction system. We speculate that in this group, systemic inflammatory processes are likely to be the major culprit. In this context, it is not surprising that longer aortic cross clamping times were a risk factor for arrhythmias in neonates. Our observation that the risk for arrhythmias became smaller with longer CPB duration is in contrast with a previous report[2] and somewhat counterintuitive. We speculate that a shorter CPB duration, achieved in part by a more rapid rewarming of the patient with shorter recirculation times, might have resulted in stronger myocardial injury in neonates.

Complete heart block is a serious complication after surgery for congenital heart disease with a reported incidence between 1 and 3%[22]. Despite a chance for late recovery, these patients carry a lower long-term survival rate and a higher incidence of sudden death[23]. The incidence of second degree AVB fell from an early peak 24 hours postoperative, but rose again to the highest postoperative incidence shortly prior to discharge. This time course may reflect a transiently improved atrioventricular conduction when initial edema resolves, followed by subacute or chronic deterioration. Interestingly, in one study, as many as 9% of the patients who had initial recovery from AVB subsequently developed second or third degree block[24].

### Limitations

The spectrum of arrhythmias in neonates may vary between institutions according to the frequencies of the various surgical interventions that are performed. For example, most of the neonates at our institution underwent an arterial switch procedure whereas Norwood or Damus-Kaye-Stansel type operations were not performed at our institution during the study era.

## Conclusions

We conclude that early postoperative arrhythmias following surgery for congenital heart disease are more frequent in infants and children than in neonates. For most types of arrhythmias surgical risk factors involving injury to a vulnerable part of the myocardial conduction system can be identified. Although overall less susceptible to postoperative arrhythmias than older children, neonates carry a higher risk of JET, especially if a VSD was closed. Postoperative arrhythmias should be anticipated in patients with arrhythmias prior to surgery.

## Competing interests

The authors declare that they have no competing interests.

## Authors' contributions

LGW: Acquisition, analysis and interpretation of data, drafting of manuscript. SK: Acquisition of data. RGG: Conception and study design, acquisition of data. BJM: Conception and study design, acquisition of data. JFV-J: Conception and study design, acquisition of data. GvB: Conception and study design, revision and final approval of manuscript. MCS: Conception and study design, acquisition of data, revision and final approval of manuscript. All authors have read and approved the final manuscript.
